# Intraoperative 3D-US-mpMRI Elastic Fusion Imaging-Guided Robotic Radical Prostatectomy: A Pilot Study

**DOI:** 10.3390/curroncol30010009

**Published:** 2022-12-22

**Authors:** Marco Oderda, Giorgio Calleris, Daniele D’Agate, Marco Falcone, Riccardo Faletti, Marco Gatti, Giancarlo Marra, Alessandro Marquis, Paolo Gontero

**Affiliations:** 1 Department of Surgical Sciences-Urology, Città della Salute e della Scienza di Torino, Molinette Hospital, University of Turin, 10126 Torino, Italy; 2 Department of Radiology, Città della Salute e della Scienza di Torino, Molinette Hospital, University of Turin, 10126 Torino, Italy

**Keywords:** elastic fusion, intraoperative, robotic radical prostatectomy, 3D, ultrasound

## Abstract

Introduction: When performing a nerve-sparing (NS) robotic radical prostatectomy (RARP), cancer location based on multiparametric MRI (mpMRI) is essential, as well as the location of positive biopsy cores outside mpMRI targets. The aim of this pilot study was to assess the feasibility of intraoperative 3D-TRUS-mpMRI elastic fusion imaging to guide RARP and to evaluate its impact on the surgical strategy. Methods: We prospectively enrolled 11 patients with organ-confined mpMRI-visible prostate cancer (PCa), histologically confirmed at transperineal fusion biopsy using Koelis Trinity. Before surgery, the 3D model of the prostate generated at biopsy was updated, showing both mpMRI lesions and positive biopsy cores, and was displayed on the Da Vinci robotic console using TilePro™ function. Results: Intraoperative 3D modeling was feasible in all patients (median of 6 min). The use of 3D models led to a major change in surgical strategy in six cases (54%), allowing bilateral instead of monolateral NS, or monolateral NS instead of non-NS, to be performed. At pathologic examination, no positive surgical margins (PSMs) were reported. Bilateral PCa presence was detected in one (9%), four (36%), and nine (81%) patients after mpMRI, biopsy, and RARP, respectively. Extracapsular extension was found in two patients (18%) even if it was not suspected at MRI. Conclusions: Intraoperative 3D-TRUS-mpMRI modeling with Koelis Trinity is feasible and reliable, helping the surgeon to maximize functional outcomes without increasing the risk of positive surgical margins. The location of positive biopsy cores must be registered in 3D models, given the rates of bilateral involvement not seen at mpMRI.

## 1. Introduction

Robot-assisted radical prostatectomy (RARP) has become the standard surgical treatment for organ-confined prostate cancer (PCa), with the aim to maximize functional recovery while maintaining oncological radicality. When feasible, a nerve-sparing (NS) approach should follow the capsular profile of the prostate to obtain the preservation of neurovascular bundles (NVBs) without incurring in positive surgical margins (PSMs) [1]. To date, the reported prevalence of PSM after RARP is approximately 9% for organ-confined disease and up to 37% for pT3 cancers, and NS surgery has been associated with an increased risk of side-specific PSM, even in low-risk cancers [2].

Currently, multiparametric magnetic resonance imaging (mpMRI) provides essential data concerning cancer location and capsular involvement and might change the extent of NS surgery in more than one out of three patients [3]. However, a non-negligible proportion of cancer foci within the gland remains unseen at mpMRI [4]. Moreover, it is still difficult for surgeons to translate mpMRI findings into real-time appreciation of tumor volume and location during RARP.

To overcome these issues, three-dimensional (3D) imaging reconstruction techniques have been proposed, including 3D printing, virtual reality, and augmented reality [5,6,7]. Indeed, 3D visualization could facilitate RARP in terms of training, surgical planning, and intraoperative guidance. However, reconstruction of 3D models is usually based on preoperative mpMRI, and therefore does not consider cancer foci not seen at mpMRI but detected with systematic sampling.

The Koelis Trinity system creates a precise 3D model of the prostate, integrating mpMRI sequences and real-time 3D ultrasound (US) with a unique elastic fusion technology that shows not only mpMRI-visible lesions, but also all positive biopsy core locations [8]. The same system can be used to perform the diagnostic fusion biopsy and to guide the execution of RARP with an intraoperative acquisition of 3D-US images to be fused with the previously generated 3D model of the prostate. The aim of this pilot study was to assess the feasibility of the intraoperative 3D-US-mpMRI elastic fusion imaging to guide RARP and to evaluate its impact on the surgical strategy to decide an NS approach.

## 2. Materials and Methods

**Figure 1:**
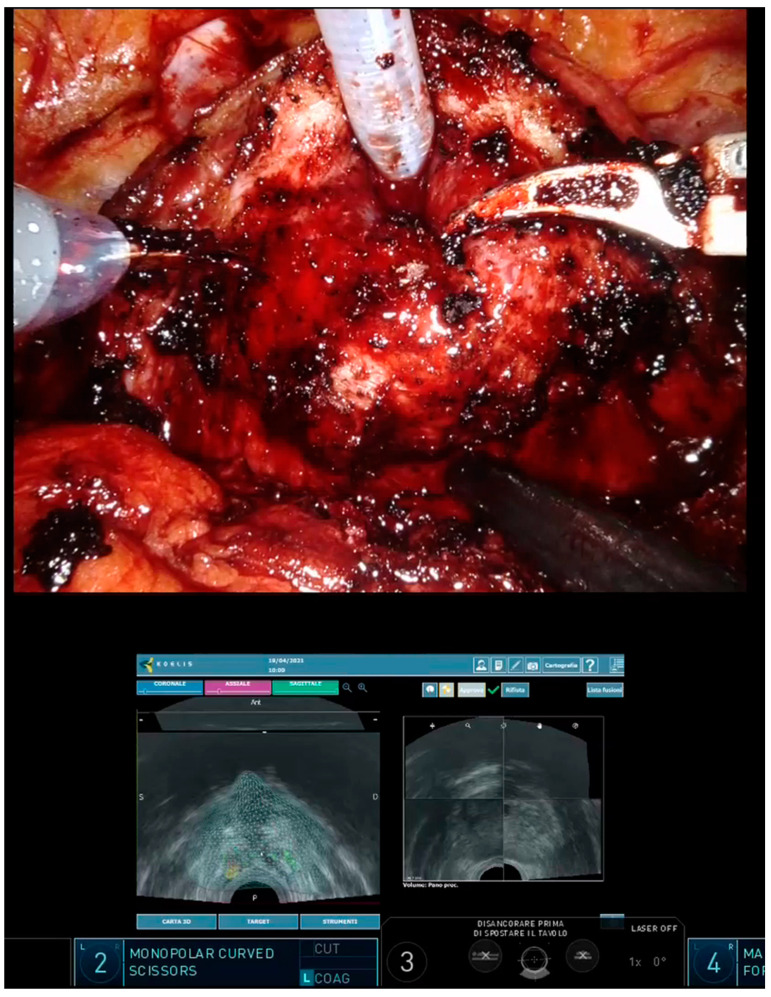
The 3D model is visualised live on the Da Vinci robotic console using the TilePro™ function, providing guidance during surgery.

This was a pilot study enrolling 11 consecutive patients addressed to RARP for clinically localized PCa. All patients had undergone diagnostic 1.5 T or 3 T mpMRI and subsequent fusion biopsy. mpMRIs were reviewed by experienced radiologists (M.G. and R.F.) and suspicious lesions were scored according to the PIRADS v2.1 classification. Fusion biopsies were performed transperineally with Koelis Trinity system (Koelis, Meylan, France), which creates a precise and highly detailed 3D map of the prostate integrating 3D-US, multimodal elastic fusion, and organ-based tracking. During the examination, a 3D transrectal US (TRUS) probe creates a 3D reference model of the prostate which is fused with mpMRI sequences showing suspicious lesions. New images are taken to register the location of the biopsy needle at each biopsy. Thanks to the organ-based technology, the device follows the position of the prostate and not that of the probe, automatically compensating for patient movement and prostate deformation. After biopsy, the 3D model of the prostate was updated with histological findings, highlighting all the biopsy cores found as PCa. On the day of RARP, all patients underwent intraoperative second-look elastic fusion imaging with Koelis Trinity; a new 3D-TRUS acquisition was performed during the initial steps of surgery (before bladder detachment), allowing for retrieval of the previous MRI and biopsy information in the current exam and the 3D model of the prostate. The output was displayed on the Da Vinci robotic console using the TilePro™ function, providing guidance during surgery (Figure 1).

RARPs were performed using a four-arm Da Vinci Xi Surgical System (Intuitive Surgical, Sunnyvale, CA, USA) by one experienced surgeon (P.G., >1000 cases), while intraoperative TRUS and fusion imaging procedures required an additional operator experienced in fusion biopsy with Koelis Trinity (M.O., >500 cases). The NS approach was defined as bilateral, unilateral, or non-NS, while the extent of NVBs preservation was defined on side-based level as intrafascial or interfascial [9].

The endpoints of the study were to evaluate the feasibility of intraoperative 3D-US-mpMRI modeling and its impact on surgical strategy as compared to the preoperative planning decided during weekly staff meetings. The pathological findings were compared to MRI and biopsy data. The study was conducted according to the Declaration of Helsinki. Ethics committee approval was waived due to non-invasive and non-interventional nature of this study. All involved patients signed an informed consent form for photo and video acquisition for clinical research purposes. Statistical analyses were performed with SPSS version 26.0 (IBM Corp, Armonk, NY, USA). The entire procedure did not lead to any additional costs so long we used the system utilized to routinely perform prostate fusion biopsies.

## 3. Results

Intraoperative 3D modeling with Koelis Trinity was feasible in all patients, requiring a very limited amount of time to be performed, with a median of 6 min per patient (range 5–10). Patients’ characteristics are shown in Table 1, including data on MRI, fusion biopsy, and radical prostatectomy.

As compared to preoperative surgical planning, the use of 3D models led to a major change in surgical strategy in six cases (54%), where a bilateral NS was performed instead of a monolateral NS, or a monolateral NS was performed instead of non-NS (Table 2).

In three cases (27%), an intrafascial NS was performed instead of an interfascial NS, thanks to the virtual localization of positive cancer cores. No change of management was reported in four patients (36%). At pathologic examination, no PSMs were reported.

All MRI targets were confirmed as PCa both at fusion biopsy and RARP. Bilateral PCa presence was detected in one patient (9%) at MRI, four patients (36%) after fusion biopsy, and nine patients (81%) after RARP. The maximum International Society of Urological Pathology (ISUP) grade at biopsy always corresponded to MRI-visible lesions. Upgrade from biopsy to RARP was detected in two patients only, from ISUP 1 to 2. Extracapsular extension was found in two patients (18%) even if it was not suspected at MRI; in both cases, the location of extracapsular extension was the same as the index lesion.

## 4. Discussion

The use of US to provide intraoperative visualization of prostatic anatomy and NVBs has been explored since 2006, when Ukimura et al. published a series of 77 patients who underwent TRUS during laparoscopic radical prostatectomy to identify NVBs, to define the prostate apex contour and to evaluate the location of hypoechoic cancer nodules [10] In their series, the use of intraoperative TRUS monitoring allowed for precise dissection tailored to the specific prostate contour anatomy, leading to a 20% decrease in PSMs [11]. More recently, the feasibility of a robotically manipulated TRUS for real-time monitoring of the prostate and periprostatic anatomy during RARP was assessed by Hung et al., showing that it can provide valuable anatomic information with the aim to maximize functional preservation [12].

While TRUS is useful in identifying real-time anatomical landmarks of the prostate, in the last few years, it has been completely replaced by mpMRI for the detection of cancer foci [13]. The integration of mpMRI and TRUS images has led to the fusion imaging that now guides most biopsies performed in the diagnostic work-up of PCa. Ukimura and Gill were the first to apply a fusion system between real-time TRUS and preoperative mpMRI during laparoscopic radical prostatectomy [14]. More recently, they developed a 3D surgical navigation model based on 3D-TRUS-guided prostate biopsies. Five key anatomic structures (prostate, image-visible biopsy-proven “index” cancer lesion, neurovascular bundles, urethra, and recorded biopsy trajectories) were image-fused and displayed onto the TilePro function of the robotic console. In their experience, the 3D model facilitated careful surgical dissection in the vicinity of the biopsy-proven index lesion, achieving negative PSMs in 90% of patients [7].

In line these authors, we believe that the potential of both 3D-TRUS and mpMRI must be exploited to build a successful, real-time 3D model of the prostate. On the strength of our experience on fusion biopsy [8], we decided to use the only available fusion system that integrates 3D-TRUS and mpMRI images, which is able to track the location of all the biopsy cores. This way, we obtained intraoperative models carrying data on the location of both mpMRI-visible and mpMRI-invisible cancer foci, detected with the systematic sampling.

In the present study, we demonstrated the feasibility of intraoperative 3D modeling with Koelis Trinity. The 3D reconstruction of the prostate was quickly obtained using the “second look” function, an option that allows for the retrieval of the 3D model constructed during the diagnostic biopsy and the location of all the biopsy cores found to be positive for PCa. We used the 3D-TRUS probe to achieve prostate volume during the first steps of surgery, before bladder detachment, to avoid image disturbances. With the transrectal probe in place, we were also able to generate several 3D models during surgery, tracking the position of the robotic instruments in relation to the location of cancer foci and identifying the exact area of the suspected pseudocapsule bulging (if any). The 3D-US-mpMRI-assisted approached allowed us to perform a nerve-sparing technique, watching out for an extracapsular extension in the exact area of risk and eventually allowing us to modify the plan of dissection. This maneuver becomes more difficult with the progress of RARP; with the development of the space between the prostate and rectum after incision of Denonvilliers’ fascia, carbon dioxide posterior to the prostate interferes with visualization. As noted by Ukimura et al., however, by this late stage of the procedure we have usually already acquired all the relevant information regarding the anatomy of the prostate and the location of cancer foci [11]. The 3D models generated by Koelis Trinity were visible at the robotic console using the TilePro™ function and were judged very helpful by the surgeon (P.G.) to visualize the location of cancer foci, especially when deciding to perform NS surgery. The guidance of 3D models led to more NS surgeries being performed, and more intrafascial approaches, as compared to what was initially planned during preoperative staff meetings, without increasing PSMs.

During this pilot study, the visualization of 3D models at the robotic console was beside the intraoperative view, without any alignment to the organ. The creation of a dedicated software able to achieve a real-time alignment of images represents the next challenge to develop automated and reliable augmented reality (AR). AR technologies have been recently proposed by Schiavina et al. [5] and Porpiglia et al. [6] with promising results to tailor the surgical dissection to the index lesions. Both models, however, were uniquely based on mpMRI images and therefore did not consider possible mpMRI-invisible locations detected by systematic sampling. Furthermore, in both cases the alignment of 3D model during surgery was manually performed by a professional, introducing a dangerously subjective element.

The comparison between pathologic and mpMRI findings in our study deserves special comment. On one hand, all lesions detected by mpMRI were confirmed to be PCa at fusion biopsy and RARP. On the other hand, however, the correlation between mpMRI findings and location of all cancerous areas in the prostate was imperfect, with a significant proportion of PCa foci found in regions other than those detected at mpMRI, even if clinically significant. Of note, the number of patients with bilateral PCa increased from one at mpMRI to four after fusion biopsy (thanks to systematic cores) and to nine after RARP. This finding is quite alarming and highlights once again the importance of a 3D model that tracks the location of positive biopsy cores. Several reports have shown the risk of finding mpMRI-invisible cancer foci. In 2019, a study performed on 185 candidates for hemiablation showed that only 33.5% of patients had unilateral cancer on final histopathology after radical prostatectomy. Significant cancer on biopsy and mpMRI-negative lobes was found in 38.9% of 185 lobes [15]. All these things considered, it would seem that mpMRI sometimes sees only the “tip of the iceberg”. In our series, the lesions detected by mpMRI were the ones with the highest grade at biopsy, and an upgrade from the biopsy to the final specimen was noted only in a minority of cases. Finally, two of our patients were diagnosed as pT3 in spite of a negative mpMRI, which can sometimes miss the initial signs of extracapsular extension. The use of 3D modeling, however, allowed us to perform more conservative surgeries without increasing our PSM rates.

We acknowledge that this is only a pilot study and further studies must be on a larger series of patients to validate these preliminary results. Furthermore, the accuracy of the 3D reconstruction should be assessed, probably with the intraoperative use of fiducials. The ultimate goal is represented by the automated alignment of the 3D model on the organ seen at the robotic console.

## 5. Conclusions

Intraoperative 3D-TRUS-mpMRI modeling with Koelis Trinity is feasible, reliable and cheap, helping surgeons to maximize functional outcomes without increasing the risk of positive surgical margins. The potential of 3D-US together with mpMRI data allows for the generation of a model that provides information on the prostate anatomy, the location of mpMRI visible cancers, and also positive systematic biopsies. The registration of biopsy cores is particularly important, given the rates of bilateral cancer involvement not seen at mpMRI.

## Figures and Tables

**Table 1:** Patients’ characteristics.

**Baseline Data**	
Age, years, mean ± SD	68.9 ± 7.4
PSA, ng/dl, mean ± SD	7.5 ± 2.1
Positive DRE, *n* (%)	4 (36%)
Previous negative biopsies, *n* (%)	4 (36%)
Prostate volume, cc, mean ± SD	44 ± 13.2
**MRI data**	
Target number, *n* (%) - Single - Multiple	8 (73%) 3 (27%)
Target location, *n* (%) - Monolateral - Bilateral	10 (91%) 1 (9%)
PIRADS score, *n* (%) - 3 - 4 - 5	1 (9%) 9 (82%) 1 (9%)
Lesion diameter, mm, mean ± SD	8.8 ± 3.5
Extracapsular extension suspicion, *n* (%)	0 (0%)
**Fusion biopsy results**	
Biopsy cores taken, *n*, median (range) - Targeted - Systematic	3 (3–6) 12 (8–20)
Cancer detection within MRI target, *n* (%)	11 (100%)
Cancer detection outside MRI target, *n* (%)	6 (54%)
Lesion location, *n* (%) - Monolateral - Bilateral	7 (64%) 4 (36%)
ISUP grade, *n* (%) - 1 - 2 - 3 - 4	2 (18%) 5 (45%) 1 (9%) 3 (27%)
ISUP upgrade due to systematic cores, *n* (%)	0 (0%)
**Radical prostatectomy findings**	
Pathological stage, *n* (%) - pT2 - pT3a	9 (81%) 2 (27%)
Positive surgical margins	0 (0%)
Cancer detection within MRI target, *n* (%)	11 (100%)
Cancer detection outside MRI target, *n* (%)	9 (82%)
ISUP grade, *n* (%) - 1 - 2 - 3 - 4	0 (0%) 7 (63%) 1 (9%) 3 (27%)
Lesion location, *n* (%) - Monolateral - Bilateral	2 (18%) 9 (82%)

DRE: digital rectal examination.

**Table 2:** Impact of 3D modeling on surgical planning and comparison between clinical and pathological findings.

	Biopsy ISUP Grade	Clinical Stage at MRI	Preoperative NS Planning	Intraoperative NS Execution	Pathological Stage	Pathological ISUP Grade
**Case 1**	2	cT2a	Monolateral NS	Bilateral NS	pT2aR0	2
**Case 2**	2	cT2a	Monolateral NS	Bilateral NS	pT2cR0	2
**Case 3**	4	cT2a	Non-NS	Non-NS	pT2bR0	4
**Case 4**	4	cT2a	Non-NS	Monolateral NS	pT2cR0	4
**Case 5**	2	cT2a	Monolateral NS	Bilateral NS	pT2cR0	2
**Case 6**	4	cT2a	Monolateral NS	Monolateral NS	pT3aR0	4
**Case 7**	1	cT2a	Bilateral NS	Bilateral NS	pT2cR0	2
**Case 8**	2	cT2a	Monolateral NS	Bilateral NS	pT2cR0	2
**Case 9**	2	cT2a	Monolateral NS	Monolateral NS	pT3aR0	2
**Case 10**	1	cT2a	Bilateral NS	Bilateral NS	pT2cR0	2
**Case 11**	3	cT2c	Monolateral NS	Bilateral NS	pT2cR0	3

## Data Availability

The data presented in this study are available on request from the corresponding author.

## Abbreviations

3D = three-dimensional; TRUS = transrectal ultrasound; mpMRI = multi-parametric magnetic resonance imaging; NS = nerve-sparing; RARP = robotic radical prostatectomy; PCa = prostate cancer; PSMs = positive surgical margins; NVB = neuro-vascular bundle.

## References

[1] Walz J., Epstein J.I., Ganzer R., Graefen M., Guazzoni G., Kaouk J., Menon M., Mottrie A., Myers R.P., Patel V. (2016). A Critical Analysis of the Current Knowledge of Surgical Anatomy of the Prostate Related to Optimisation of Cancer Control and Preservation of Continence and Erection in Candidates for Radical Prostatectomy: An Update. Eur. Urol..

[2] Yossepowitch O., Briganti A., Eastham J.A., Epstein J., Graefen M., Montironi R., Touijer K. (2014). Positive Surgical Margins After Radical Prostatectomy: A Systematic Review and Contemporary Update. Eur. Urol..

[3] Marenco J., Orczyk C., Collins T., Moore C., Emberton M. (2019). Role of MRI in planning radical prostatectomy: What is the added value?. World J. Urol..

[4] Bonekamp D., Schelb P., Wiesenfarth M., Kuder T.A., Deister F., Stenzinger A., Nyarangi-Dix J., Röthke M., Hohenfellner M., Schlemmer H.-P. (2019). Histopathological to multiparametric MRI spatial mapping of extended systematic sextant and MR/TRUS-fusion-targeted biopsy of the prostate. Eur. Radiol..

[5] Schiavina R., Bianchi L., Lodi S., Cercenelli L., Chessa F., Bortolani B., Gaudiano C., Casablanca C., Droghetti M., Porreca A. (2020). Real-time Augmented Reality Three-dimensional Guided Robotic Radical Prostatectomy: Preliminary Experience and Evaluation of the Impact on Surgical Planning. Eur. Urol. Focus.

[6] Porpiglia F., Checcucci E., Amparore D., Manfredi M., Massa F., Piazzolla P., Manfrin D., Piana A., Tota D., Bollito E. (2019). Three-dimensional Elastic Augmented-reality Robot-assisted Radical Prostatectomy Using Hyperaccuracy Three-dimensional Reconstruction Technology: A Step Further in the Identification of Capsular Involvement. Eur. Urol..

[7] Ukimura O., Aron M., Nakamoto M., Shoji S., Abreu A.L.D.C., Matsugasumi T., Berger A., Desai M., Gill I.S. (2014). Three-dimensional surgical navigation model with TilePro display during robot-assisted radical prostatectomy. J. Endourol..

[8] Oderda M., Marra G., Albisinni S., Altobelli E., Baco E., Beatrici V., Cantiani A., Carbone A., Ciccariello M., Descotes J.-L. (2018). Accuracy of elastic fusion biopsy in daily practice: Results of a multicenter study of 2115 patients. Int. J. Urol. Off. J. Jpn. Urol. Assoc..

[9] Walz J., Burnett A.L., Costello A.J., Eastham J.A., Graefen M., Guillonneau B., Menon M., Montorsi F., Myers R.P., Rocco B. (2010). A critical analysis of the current knowledge of surgical anatomy related to optimization of cancer control and preservation of continence and erection in candidates for radical prostatectomy. Eur. Urol..

[10] Ukimura O., Gill I.S. (2006). Real-time transrectal ultrasound guidance during nerve sparing laparoscopic radical prostatectomy: Pictorial essay. J. Urol..

[11] Ukimura O., Magi-Galluzzi C., Gill I.S. (2006). Real-time transrectal ultrasound guidance during laparoscopic radical prostatectomy: Impact on surgical margins. J. Urol..

[12] Hung A.J., Abreu A.L.D.C., Shoji S., Goh A.C., Berger A.K., Desai M.M., Aron M., Gill I.S., Ukimura O. (2012). Robotic transrectal ultrasonography during robot-assisted radical prostatectomy. Eur. Urol..

[13] Mottet N., van den Bergh R.C., Briers E., Van den Broeck T., Cumberbatch M.G., De Santis M., Fanti S., Fossati N., Gandaglia G., Gillessen S. (2021). EAU-EANM-ESTRO-ESUR-SIOG Guidelines on Prostate Cancer-2020 Update. Part 1: Screening, Diagnosis, and Local Treatment with Curative Intent. Eur. Urol..

[14] Ukimura O., Ahlering T.E., Gill I.S. (2008). Transrectal ultrasound-guided, energy-free, nerve-sparing laparoscopic radical prostatectomy. J. Endourol..

[15] Choi Y.H., Yu J.W., Kang M.Y., Sung H.H., Jeong B.C., Seo S.I., Jeon S.S., Lee H.M., Jeon H.G. (2019). Combination of multiparametric magnetic resonance imaging and transrectal ultrasound-guided prostate biopsies is not enough for identifying patients eligible for hemiablative focal therapy for prostate cancer. World J. Urol..

